# Long-Term Cardiac Damage Associated With Abdominal Irradiation in Mice

**DOI:** 10.3389/fphar.2022.850735

**Published:** 2022-02-22

**Authors:** Zhaojia Wang, Ziheng Jia, Zandong Zhou, Xiaotong Zhao, Feng Wang, Xu Zhang, Gary Tse, Guangping Li, Yang Liu, Tong Liu

**Affiliations:** ^1^ Tianjin Key Laboratory of Ionic-Molecular Function of Cardiovascular Disease, Department of Cardiology, Tianjin Institute of Cardiology, Second Hospital of Tianjin Medical University, Tianjin, China; ^2^ Institute of Radiation Medicine, Chinese Academy of Medical Sciences & Peking Union Medical College, Tianjin, China; ^3^ Department of Genetics, School of Basic Medical Sciences, Tianjin Medical University, Tianjin, China; ^4^ Tianjin Key Laboratory of Metabolic Diseases, Collaborative Innovation Center of Tianjin for Medical Epigenetics, Center for Cardiovascular Diseases, Research Center of Basic Medical Sciences, Department of Physiology and Pathophysiology, Tianjin Medical University, Tianjin, China; ^5^ Kent and Medway Medical School, Canterbury, United Kingdom

**Keywords:** irradiation, cardiac function, cardiac remodeling, metabolomics, bystander effect

## Abstract

**Aims:** Irradiation is an effective treatment for tumors but has been associated with cardiac dysfunction. However, the precise mechanisms remain incompletely elucidated. This study investigated the long-term cardiac damage associated with abdominal irradiation and explored possible mechanisms.

**Methods and Results:** Wild-type C57BL6/J mice were divided into two groups: untreated controls (Con) and treatment group receiving 15 Gy of abdominal gamma irradiation (AIR). Both groups received normal feeding for 12 months. The AIR group showed reductions in left ventricular ejection fraction (LVEF), fractional shortening (FS), left ventricular end-diastolic internal diameter (LVID; d), left ventricular end-diastolic volume (LV Vol. diastolic volume (LV Vol; d) and mitral transtricuspid flow late diastolic filling velocity (MV A). It also showed increased fibrosis, reduced conduction velocity and increased conduction heterogeneity. Non-targeted metabolomics showed the differential metabolites were mainly from amino acid metabolism. Further KEGG pathway annotation and enrichment analysis revealed that abnormalities in arginine and proline metabolism, lysine degradation, d-arginine and d-ornithine metabolism, alanine, aspartate and glutamate metabolism, and arginine biosynthesis.

**Conclusion:** Abdominal irradiation causes long-term damage to the non-irradiated heart, as reflected by electrical and structural remodeling and mechanical dysfunction associated with abnormal amino acid biosynthesis and metabolism.

## Introduction

Cancer is the leading cause of mortality worldwide (Soerjomataram and Bray, 2021; Sung et al., 2021). Approximately 19.3 million new cancer diagnoses are made whilst 10 million cancer-deaths are observed each year globally. Due to the early diagnosis and timely intervention of cancer treatment in recent years, deaths due to cancer itself have significantly receded, while cardiac disease and damage caused by tumor treatment have clearly become a major cause of non-neoplastic death among cancer survivors (Curigliano et al., 2016; Armanious et al., 2018; Li H. et al., 2019; Alvarez-Cardona et al., 2020). Irradiation is an important treatment for cancer. However, it can exert adverse effects on normal and otherwise healthy tissues and organs, with significant negative impact on prognosis of cancer patients (Schaue and McBride, 2015; Nielsen et al., 2017; Chargari et al., 2019). For example, its related cardiac damage occurs in both short- and long-term (Groarke et al., 2014; Cao et al., 2021), while the early stages of cardiac dysfunction may be asymptomatic.

Long-term cardiac damage includes valvular heart disease, coronary heart disease, arrhythmia, pericardial stenosis, heart failure (Simone, 2017; Menezes et al., 2018; Nabialek-Trojanowska et al., 2020). Therefore, there is a need to better clarify the specific mechanisms underlying cardiac damage. The degree of cardiac damage is usually greater with a closer irradiation window and higher doses applied, as reflected by radiotherapy-related cardiac damage in the treatment of thoracic tumors such as breast cancer, lung cancer, and lymphoma (Bradley et al., 2015; Gkantaifi et al., 2019; Banfill et al., 2021).

By contrast, there are fewer reports of cardiac damage related to irradiation that do not directly target the heart. Previous studies have shown that oxidative stress in the heart after craniocerebral irradiation may be related to the bystander effect (Rashed et al., 2021). However, it is unclear whether other parts of the irradiation will also produce toxic effects on the heart directly. Clinical studies have reported that radiotherapy for peptic ulcer is a risk factor for coronary heart disease which can explain radiation-related cardiac dysfunction (Carr et al., 2005). Abdominal cancers are common (Sung et al., 2021) but there have been few mechanistic studies on the relationship between abdominal irradiation, possible cardiac dysfunction and the underlying mechanisms. Therefore, the aim of this study is to explore the long-term adverse effects on the heart caused by abdominal irradiation in a mouse model.

## Materials and Methods

### Experimental Animal Models and Ethical Approval

All animal experiments were approved by the Institutional Committee for the Care and Use of Animals, Institute of Radiological Sciences, Chinese Academy of Medical Sciences (approval number: IRM-DWLL-2019149). Pathogen-free male C57BL6/J mice (6–8-week-old) were purchased from Beijing Vital River Laboratory Animal Technology Company. All the mice were equally divided into control group and abdominal irradiation group. Mice in irradiation group received a single dose of 15 Gy abdominal irradiation in a Gammacell-40^137^Cesium gray irradiator (Atomic Energy of Canada Ltd., Chalk River, Ontario, Canada). All mice were subjected to echocardiographic, electrophysiological and metabolomic analyses at 12 months post-irradiation along with control mice. All the mice were fed in a standard environment (20 ± 1°C room temperature, 50 ± 10% relative humidity) for 12 h of light and dark cycling with ad libitum diet and water. For sampling, all mice were euthanised using anaesthetic exposure.

### Echocardiography

Echocardiography was performed using the Vevo 2,100 high-resolution ultrasound imaging system. Isoflurane anaesthesia was used for supine fixation. Left ventricular end-diastolic internal diameter (LVEDD), left ventricular end-systolic internal diameter (LVESD), left ventricular end-diastolic volume (LV Vol; d) and left ventricular end-systolic volume (LV Vol; s) were measured and recorded in the short-axis view of the left heart. Left ventricular ejection fraction (LVEF) and shortening fraction (FS) were calculated as indicators of LV systolic function. In the apical four-chamber view, a sampling frame was placed at the mitral orifice and the left ventricular outflow tract to monitor mitral *trans*-tricuspid flow, early diastolic filling velocity (E) and late diastolic filling velocity (A), allowing quantification of the E/A ratio, reflecting diastolic function. Five different cardiac cycles were measured for each assessment.

### Epicardial Electrogramming

Epicardial electrical activity was recorded by placing 6 x 6 electrode microelectrodes (electrode impedance: 1.5–1.7Q, PA03606060101, multi-electrode probe array) on the epicardial surface. Data were recorded by a multichannel system (EMS64-USB-1003, United Kingdom). The obtained activation waveforms were amplified by a filter amplifier and thus transmitted to a computer. All activation times were digitised and used to plot activation maps. The activation time is calculated as the point of maximum negative slope of the activation waveform. Conduction velocity (CV), inhomogeneity index and absolute inhomogeneity were calculated by EMapScope 4.0 software (MappingLab Ltd., United Kingdom).

### Histological Analysis

For hematoxylin-eosin (HE) staining, paraffin-embedded tissues were dewaxed and hematoxylin stained for 3–8 min, followed by alcoholic fractionation with 1% hydrochloric acid for a few seconds and final immersion in eosin staining solution for 1–3 min. For Masson staining, dewaxed tissue sections were immersed in Weiger iron hematoxylin stain for 10 min. After grading with 0.5% hydrochloric acid ethanol for 15 s, the sections were stained with lychee red acid magenta solution for 8 min. This was followed by treatment with 1% phosphomolybdic acid in water for approximately 5 min and direct staining with aniline blue solution or bright green solution for a further 5 min. Finally, all sections were treated with 1% glacial acetic acid for 1 min. Relative quantitative analysis of the fibrosis in Masson stains is the mean of the six field positive area ratios (positive area divided by total area).

### Non-Targeted Metabolomics Sample Preparation

15 mg of tissue was used for grinding (50 Hz, 5 min), followed by sonication in a water bath at 4°C for 10 min, and then left to stand for 1 h in a refrigerator at -20°C. The tissue was centrifuged at 25,000 rcf for 15 min at 4°C. After centrifugation, 600 μl of supernatant is placed in a cryogenic vacuum concentrator and 200 μl of reagent solution (acetonitrile: H2O = 7:3, v: v) is added to redissolve. The samples were vortex-shocked for 1 min, sonicated in a water bath at 4°C for 10 min, centrifuged at 25, 000 rcf for 15 min at 4°C and the supernatant was placed in a supernatant bottle. 20 μl of supernatant from each sample was mixed into QC quality control samples and used to assess the reproducibility and stability of the LC-MS analytical process.

### Non-Targeted Metabolomics Analysis

LC-MS/MS technology was used for untargeted metabolomics analysis, using a high-resolution mass spectrometer Q Exactive (Thermo Fisher Scientific, United States) to collect data from positive and negative ions to improve metabolite coverage. LC-MS/MS data processing was performed using The Compound Discoverer 3.1 (Thermo Fisher Scientific, United States) software, which primarily included peak extraction, peak alignment and compound identification. Data pre-processing, statistical analysis, metabolite taxonomic annotation and functional annotation were performed using the metabolomics R package metaX and metabolome bioinformatics analysis pipeline developed by UW Genetics. The multivariate raw data were downscaled by PCA (Principal Component Analysis) to analyse the grouping, trends (within and between group similarities and differences) and outliers (presence of outlier samples) of the observed variables in the dataset. Using PLSDA (Partial Least Squares - Discriminant Analysis), VIP (Variable Importance in Projection) values for the first two main components of the model were combined with analysis of variability, ploidy change and student tests to screen for differential metabolites.

### Statistical Analysis

Differences were considered statistically significant if *p* < 0.05 (*). Significance of differences between groups was analysed using t-tests. All data were analysed using SPSS 17.0.

## Results

### Abdominal Radiation-Related Damage on Ventricular Structure and Function

**FIGURE 1:**
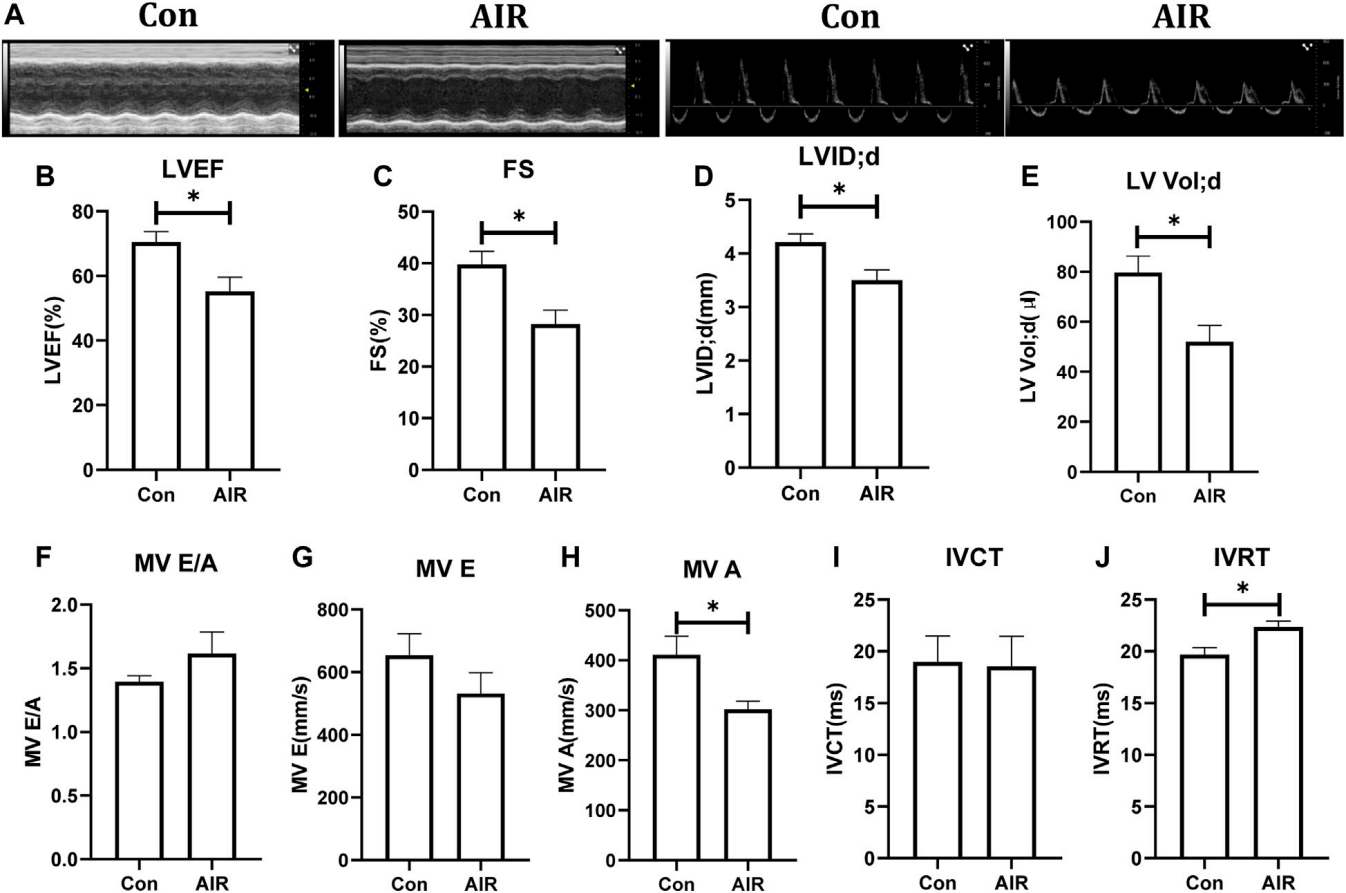
Changes in cardiac function in mice with or without abdominal radiation. **(A)** Echocardiography shows in short-axis view (left) and apical four-chamber view (right). **(B)** Left ventricular ejection fraction (LVEF) (%). **(C)** Fractional shortening (FS) (%). **(D)** Left ventricular end-diastolic internal diameter (LVID; d). **(E)** Left ventricular end-diastolic volume (LV Vol; d). **(F)** Ratio of mitral transtricuspid flow early diastolic filling velocity and late diastolic filling velocity (MV E/A) **(G)** Mitral transtricuspid flow early diastolic filling velocity (MV E). **(H)** Mitral transtricuspid flow late diastolic filling velocity (MV A). **(I)** Isovolumic contraction time (IVCT). **(J)** Isovolumic relaxation time (IVRT). n = 5, *p < 0.05.

In the initial experiments, cardiac mechanical function and structure were examined using echocardiography. Left ventricular ejection fraction (LVEF), fractional shortening (FS), left ventricular end-diastolic internal diameter (LVID; d), left ventricular end-diastolic volume (LV Vol; d) and mitral transtricuspid flow late diastolic filling velocity (MV A) were reduced and isovolumic relaxation time (IVRT) was increased in the abdominal radiation group compared to the control group. Other parameters, such as mitral transtricuspid flow early diastolic filling velocity (MV E) and the ratio of mitral transtricuspid flow early diastolic filling velocity and late diastolic filling velocity (MV E/A) showed a tendency to deteriorate but the effects were not statistically significant (Figures 1A–J).

**FIGURE 2:**
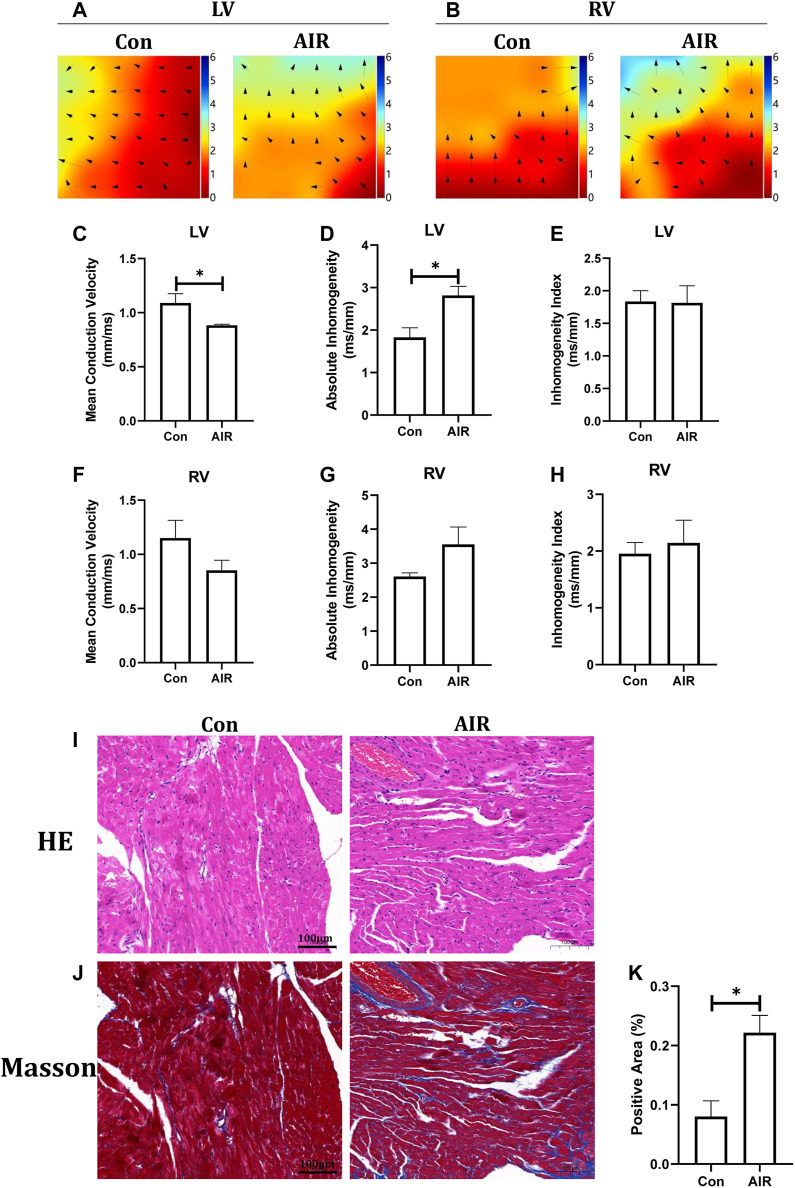
Changes in ventricular electrical and structural remodeling in mice with or without abdominal radiation. Color-coded simulated epicardial activation map of with or without abdominal radiation in left ventricles **(A)** and right ventricles **(B)**. Mean epicardial electrical conduction velocity **(C)**, absolute inhomogeneity **(D)** and inhomogeneity index **(E)** of mice with or without abdominal radiation in left ventricles. Mean epicardial electrical conduction velocity **(F)**, absolute inhomogeneity **(G)** and inhomogeneity index **(H)** of mice with or without abdominal radiation in right ventricles. H&E stains **(I)** and Masson stains **(J)** of ventricle **(K)**. Relative quantitative analysis of the fibrosis in Masson stains of ventricle. The scale bar is 100 μm. n = 5, *p < 0.05.

Electrical remodeling of the ventricles was evaluated using epicardial optical mapping. The mean electrical conduction velocity of the left ventricles was decreased and the absolute inhomogeneity, one of parameters of conduction heterogeneity, was increased in abdominal radiation group, suggesting an abnormal electrical conduction in the left ventricles. By contrast, the mean conduction velocity and heterogeneity of the right ventricles did not show significant changes (Figures 2A–H). Further tissue level studies using H&E stains showed no significant infiltration by inflammatory cells in abdominal radiation group (Figure 2I). Masson stain showed increased positive area of intramyocardial fibrosis, which could indicate ventricular structural remodeling in abdominal radiation group (Figures 2J,K).

### Abdominal Radiation-Related Changes on Ventricular Small Molecule Metabolites

**FIGURE 3:**
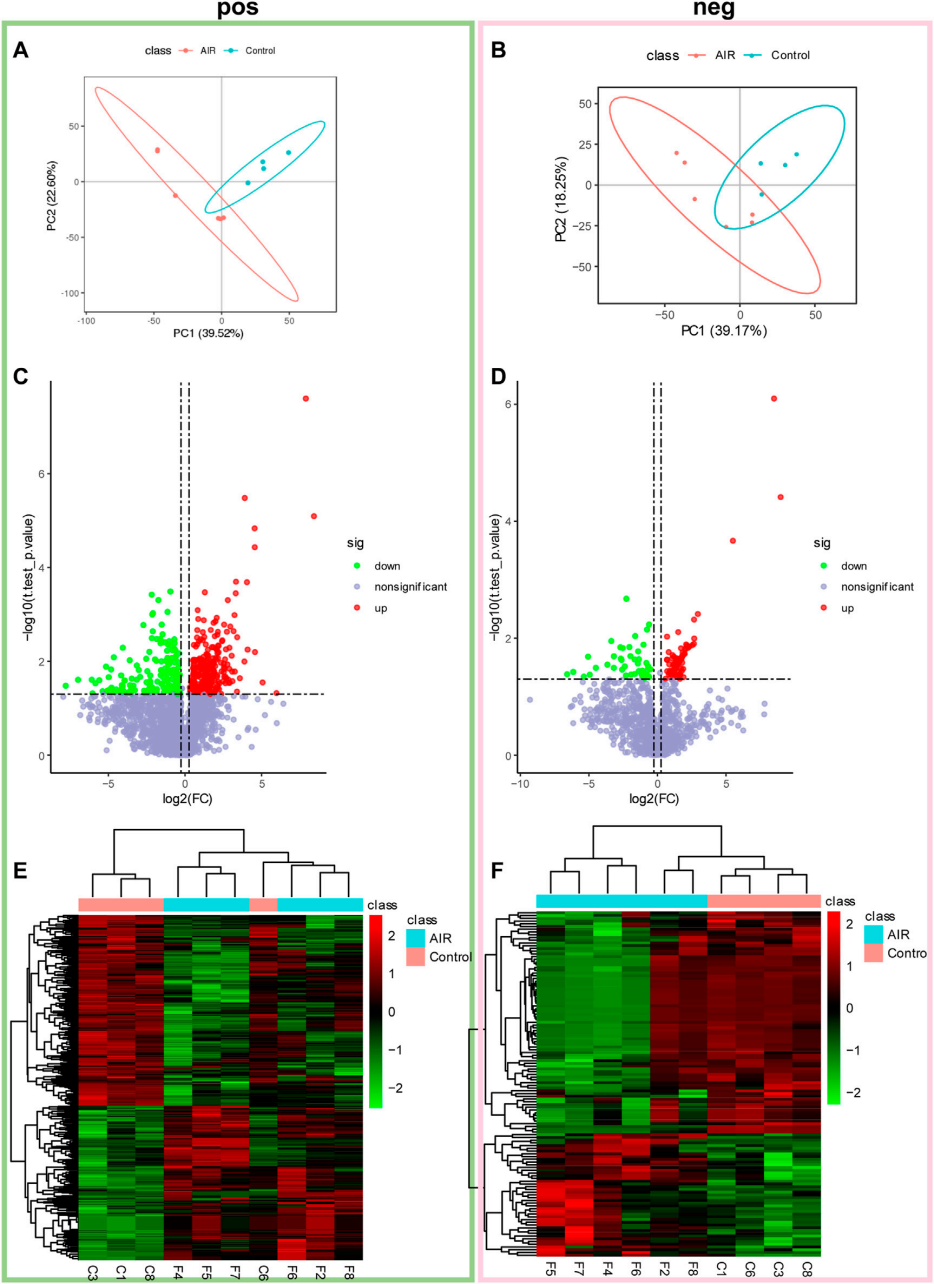
Abdominal radiation significantly changes the tissue metabolic profile of ventricles. Extracted metabolites were analyzed in positive and negative modes as described. Partial least square-discriminant analysis (PLS-DA) and volcano plots of detected metabolites in positive mode and negative mode. In positive mode **(A)** and negative mode **(B)**, 2D scores plot shows PLS-DA discrimination between samples from ventricles of abdominal radiation group (AIR, red) and controls group (Control, blue). The shaded areas indicate the 95% confidence regions. Volcano plots show metabolites from ventricles in control group compared to abdominal radiation group in positive mode **(C)** and negative mode **(D)**. Significantly differential metabolites are highlighted in red (upregulated) and green (downregulated). Heatmap of the significantly altered metabolites between control and abdominal radiation group in positive mode **(E)** and negative mode **(F)**. Before cluster analysis, the data is processed by Log2 transformation and zero-mean normalization. The cluster analysis uses the PHEATMap function in the PHEATMap R package.

Subsequently, non-targeted metabolomics analysis was used to identify small molecular metabolites changes. Multiple variance statistical analysis (PCA and PLS-DA) and single variance analysis (folding variation (FC) and student tests) were used to identify changes in the levels of metabolites. PLS-DA of metabolites showed a distinct separation between abdominal radiation and control groups (Figures 3A,B). In the positive mode, a total of 474 metabolites were differentially expressed. Amongst these, 262 metabolites were up-regulated and 212 were down-regulated in control group compared to the abdominal radiated group. In the negative mode, 126 metabolites were significantly differentially expressed; among them, 81 metabolites were up-regulated and 45 were down-regulated in control group compared to the radiated samples (Figures 3C–F).

**FIGURE 4:**
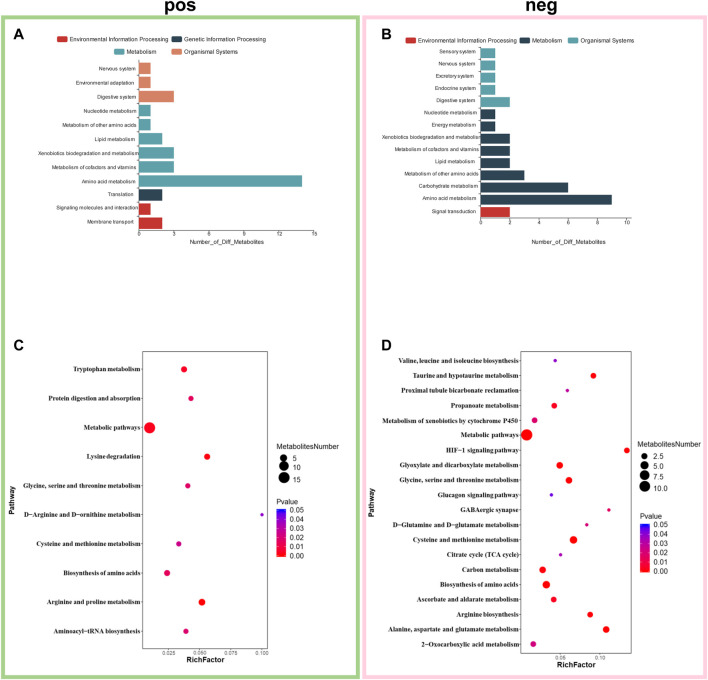
Enrichment of differential metabolites and pathways. Classification and annotation of identified differential metabolites to in positive mode **(A)** and negative mode **(B)**. Metabolic pathway enrichment analysis in positive mode **(C)** and negative mode **(D)**. *p < 0.05, fold change≥1.2 or ≤0.83.

**FIGURE 5:**
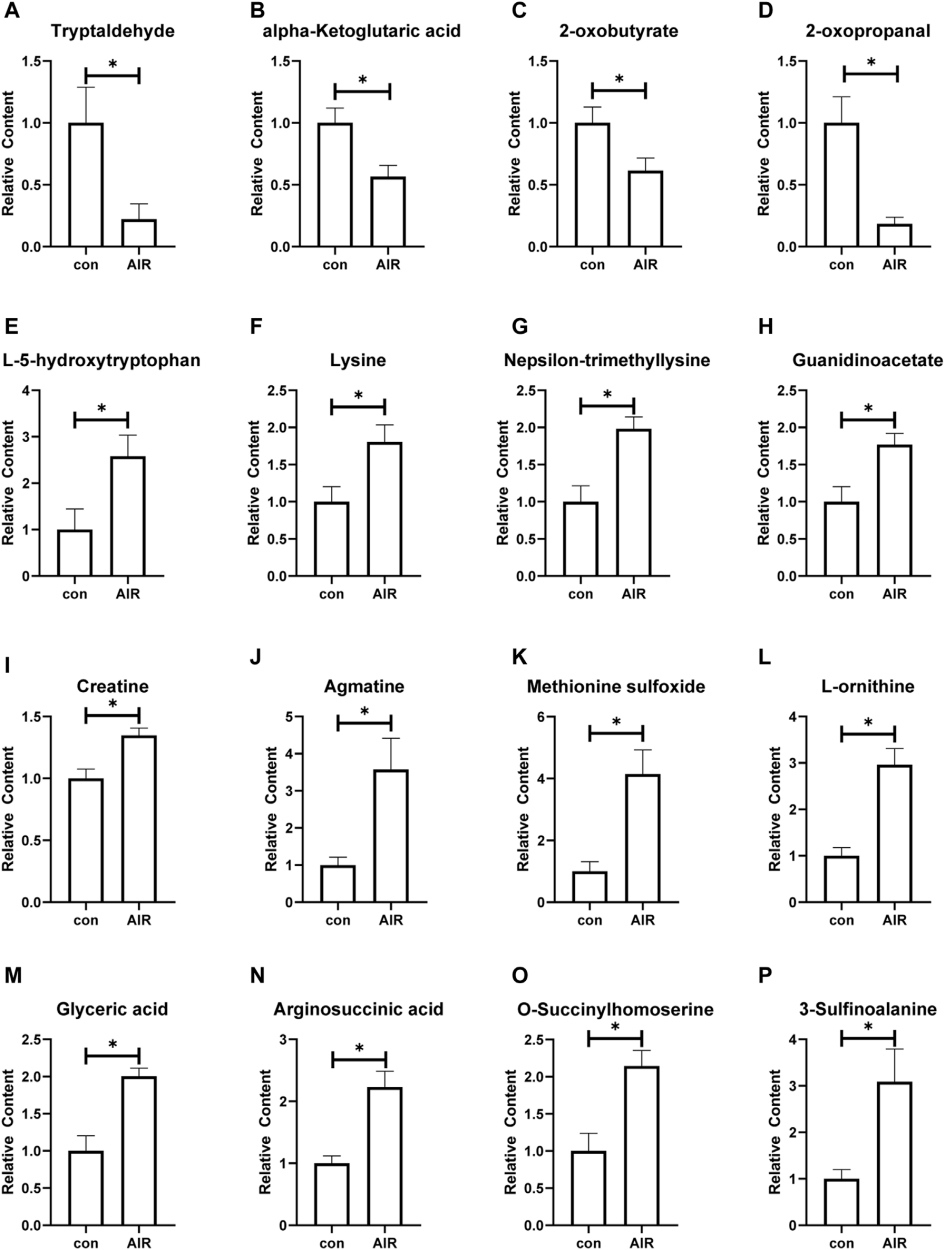
Changes of amino acid metabolites of ventricles in mice with or without abdominal radiation. Relative content of tryptaldehyde **(A)**, alpha-ketoglutaric acid **(B)**, 2-oxobutyrate **(C)**, 2-oxopropanal **(D)**, l-5-hydroxytryptophan **(E)**, lysine **(F)**, nepsilon-trimethyllysine **(G)**, guanidinoacetate **(H)**, creatine **(I)**, agmatine **(G)**, methionine sulfoxide **(K)**, l-ornithine **(L)**, glyceric acid **(M)**, arginosuccinic acid **(N)**, *o*-succinylhomoserine **(O)**, 3-sulfinoalanine **(P)**. n = 4–6, *p < 0.05.

The KEGG and HMDB databases were used to classify and annotate the metabolites that were significantly different between the abdominal radiation and control groups. The identified differential metabolites were mainly concentrated in amino acid metabolism and carbohydrate metabolism (Figures 4A,B). Changes in amino acid metabolites are as follows: tryptaldehyde, alpha-ketoglutaric acid, 2-oxobutyrate, 2-oxopropanal decreased, while l-5-hydroxytryptophan, lysine, nepsilon-trimethyllysine, guanidinoacetate, creatine, agmatine, methionine sulfoxide, l-ornithine, glyceric acid, arginosuccinic acid, *o*-succinylhomoserine, 3-sulfinoalanine increasing (Figure 5).

Metabolic pathway enrichment analysis of the identified differential metabolites revealed that they were mainly enriched in arginine and proline metabolism, lysine degradation, tryptophan metabolism, biosynthesis of amino acids, protein digestion and absorption, glycine, serine and threonine metabolism, aminoacyl-tRNA biosynthesis, cysteine and methionine metabolism, d-arginine and d-ornithine metabolism, cysteine and methionine metabolism, alanine, aspartate and glutamate metabolism, biosynthesis of amino acids, glycine, serine and threonine metabolism, glyoxylate and dicarboxylate metabolism, HIF-1 signaling pathway, taurine and hypotaurine metabolism, arginine biosynthesis, carbon metabolism, propanoate metabolism, ascorbate and aldarate metabolism, gabaergic synapse, metabolism of xenobiotics by cytochrome P450, d-glutamine and d-glutamate metabolism and 2-Oxocarboxylic acid metabolism (Figures 4C,D).

### Abdominal Radiation-Related Damage on Atrial Structure

**FIGURE 6:**
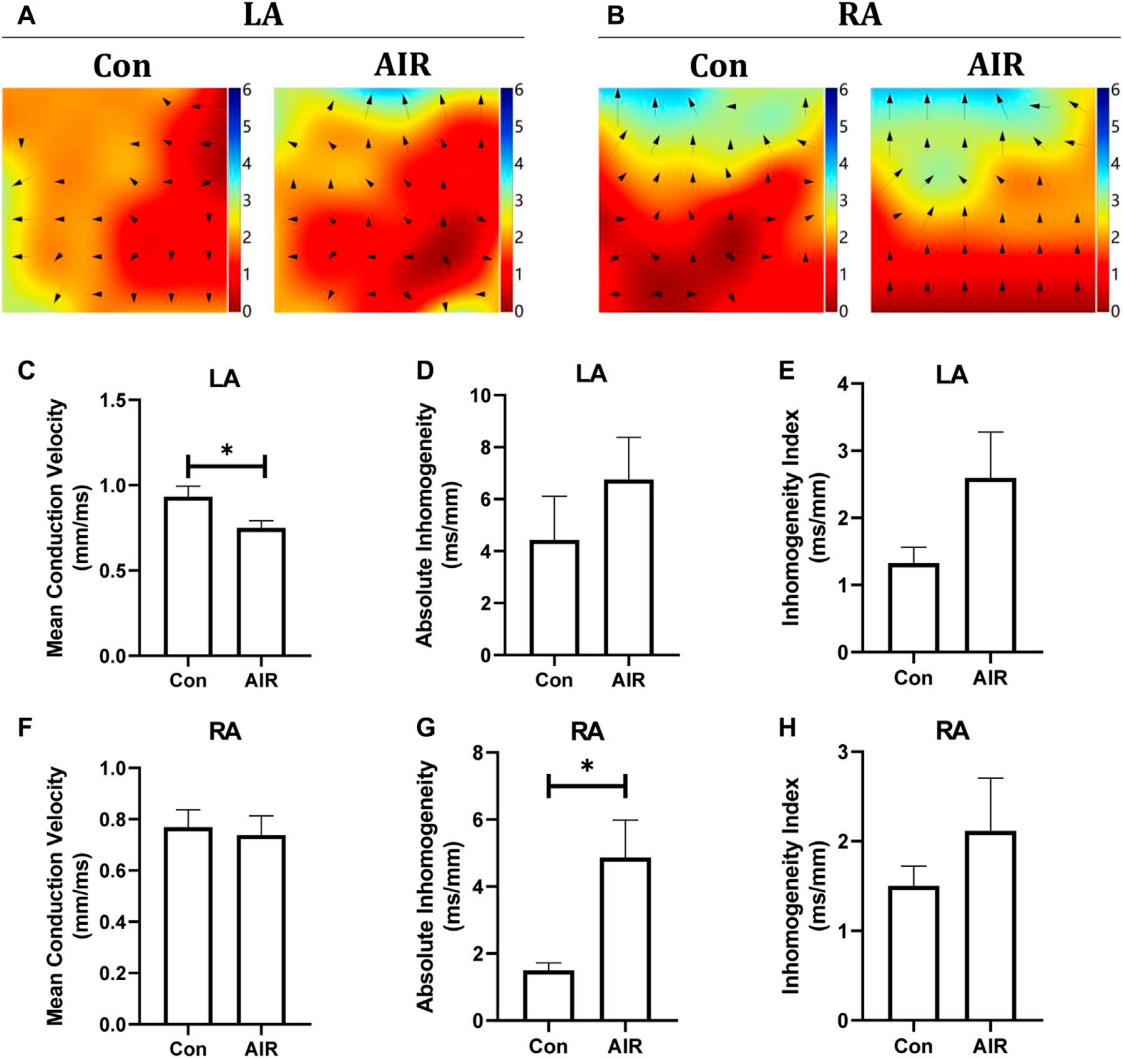
Changes in cardiac electrical conduction of mice with or without abdominal radiation. Color-coded simulated epicardial activation map of mice in left atrium **(A)** and right atrium **(B)** with or without abdominal radiation. Mean epicardial electrical conduction velocity **(C)**, absolute inhomogeneity **(D)** and inhomogeneity index **(E)** of mice with or without abdominal radiation in left atrium. Mean epicardial electrical conduction velocity **(F)**, absolute inhomogeneity **(G)** and inhomogeneity index **(H)** of mice with or without abdominal radiation in right atrium. H&E stains (I) and Masson stains (J) of atrium. The scale bar is 100 μm.n = 5, *p < 0.05.

The effects on structural and electrical remodelling in the atria were also examined. Epicardial optical mapping showed that the mean electrical conduction velocity of the left atrium was decreased, and the absolute inhomogeneity of the right atrium was increased in abdominal radiation group compared to control group (Figures 6A–H). By contrast, the absolute inhomogeneity of the left atrium and inhomogeneity index of the both left and right atrium trended to increase but the changes were not statistically significant. Similar to the findings in the ventricles, HE stains and Masson stains showed no significant infiltration by inflammatory cells and intramyocardial fibrosis in abdominal radiation group (**data not shown**).

## Discussion

In this study, long-term cardiac damage caused by abdominal irradiation in mice were examined. Cardiac mechanical dysfunction, associated with electrical and structural remodeling, was observed. These changes were associated with abnormalities in amino acid biosynthesis and metabolism.

The resultant cardiac damage by abdominal irradiation can be due to a number of reasons. It could be the bystander effect of radiotherapy, that is, the same effect of radiotherapy on cells, tissues or organs in non-irradiated areas (Wang et al., 2015; Fu et al., 2020). After irradiation, cells in the irradiated area can secrete cytokines, small molecular metabolites, exosomes or microvesicles containing various substances, and other media (Lin et al., 2017; Ariyoshi et al., 2019; Ni et al., 2020; Mukherjee et al., 2021), resulting in metabolic disorders, apoptosis, endoplasmic reticulum and golgi dysfunction of cells in non-irradiated areas (Yakovlev, 2015; Jella et al., 2018; Mladenov et al., 2018; Dong et al., 2020; Heeran et al., 2021; Yang et al., 2021). Earlier studies reported elevated levels of CRP in circulating blood, and splenic cells exhibited enhanced levels of oxidative stress and increased apoptosis after cranial irradiation in rats (El-Din et al., 2017). Isolated extracellular vesicles in the circulation of systemically irradiated mice were injected into unirradiated mice, resulting in increased levels of lipid peroxidation and reactive oxygen species in the spleen of bystander mice with spleen damage (Hargitai et al., 2021). However, the bystander effect of abdominal irradiation on the heart is still unclear. This study clarified that irradiation of the abdomen in mice can cause long-term damage to the structure and function of the heart in the non-irradiated position, which may be mediated by small molecular metabolites.

Several pathophysiological processes, such as myocardial fibrosis, atherosclerosis, microvascular and macrovascular ischemia, underlie the cardiac damage from irradiation. The possible mechanisms are also diverse, such as vascular damage, inflammatory response, platelet activation, calcium overload, endoplasmic reticulum and mitochondrial oxidative stress and autonomic dysfunction (Yusuf et al., 2017; Us et al., 2020; Zhang et al., 2020). Recent studies have shown that mRNA levels of inflammasome-associated chemokines CCL2 and CCL5 and the adhesion molecule VCAM1 were significantly higher after direct radiation treatment of arteries compared to controls, suggesting that radiation-induced inflammatory factors play an important role in adverse cardiovascular effects (Christersdottir et al., 2019). We speculate that inflammatory factors from the radiotherapy site may enter the heart through the blood circulation and cause heart damage. In the rat model, it was found that the levels of inflammatory factors such as IL-4, IL-6, IL1-β and TNF-α and oxidative stress-related factors such as Malondialdehyde were significantly increased (Us et al., 2020). IL-6 acts on the gp130/STAT3 pathway to cause cardiomyocyte hypertrophy to provide a prerequisite for heart failure (Meléndez et al., 2010). TNF-α-mediated intracellular calcium overload leads to myocardial electrical activity and contractile dysfunction (Hanna and Frangogiannis, 2020). In addition, immune checkpoint inhibitors may increase the risk of radiation myocardial toxicity due to the fact that radiation myocardial toxicity may be mediated by the PD-1 axis and CD8-positive T cells (Du et al., 2018). This may be the reason for the changes in the function and structure of the cardiac changes in our animal model. In addition, heart damage related to craniocerebral radiotherapy may be related to oxidative stress (Rashed et al., 2021). This is consistent with the abnormal results of small molecule metabolites in our model.

Our study found abnormal amino acid metabolism of myocardial tissue in the abdominal radiation group. The metabolome results showed the decreased tryptaldehyde, alpha-ketoglutaric acid, 2-oxobutyrate, 2-oxopropanal and the increased l-5-hydroxytryptophan, lysine, nepsilon-trimethyllysine, guanidinoacetate, creatine, agmatine, methionine sulfoxide, l-ornithine, glyceric acid, arginosuccinic acid, *o*-succinylhomoserine, 3-sulfinoalanine increasing based on the findings. Kyoto Encyclopedia of Genes and Genomes database (https://www.genome.jp/kegg/) and Human Metabolome database (https://hmdb.ca/), we have analyzed the effect of different metabolite changes on the heart. Alpha-Ketoglutaric acid participates in the tricarboxylic acid cycle and eventually causes abnormal energy metabolism. It is also involved in metabolism of a variety of amino acids, such as alanine, aspartic acid and glutamic acid metabolism, lysine biosynthesis, histidine metabolism and so on. 2-oxobutyrate is a substance involved in the metabolism of a variety of amino acids (glycine, methionine, valine, leucine, serine, threonine, isoleucine), as well as the metabolism of propionic acid and C-5 branched-chain dibasic acid. It is also one of the degradation products of threonine. It can be converted into propionyl-CoA (and subsequently methylmalonyl-CoA, which can be converted into succinyl-CoA, a citric acid cycle intermediate), and thus enter the citric acid cycle. In addition, valine, leucine and isoleucine were also biosynthesized by propionic acid and C-5 branched-chain dibasic acid. 2-oxopropanal participates in the metabolism of glycine, serine and threonine, pyruvate metabolism, and propionic acid metabolism. Tryptaldehyde is involved in many enzymatic reactions. Therefore, when the content of the above substances is significantly reduced, it will not only reduce the energy supply of the heart, but also affect the synthesis and metabolism of the normal amino acids of the myocardium, and ultimately affect the changes in structure and function.

O-succinylhomoserine, 3-sulfinoalanine, and methionine sulfoxide are involved in the metabolism of cysteine and methionine metabolism. In this model, the content of O-succinylhomoserine, 3-sulfinoalanine, and methionine sulfoxide in the abdominal irradiation group all increased significantly, suggesting that the metabolism of cysteine or methionine was enhanced. N-acetylcysteine exerts a strong antioxidant effect on myocardial protection (Johnson et al., 2019; Mushtaq et al., 2021), the decrease of which may suggest that the model mouse myocardium may have reduced antioxidant capacity. Glyceric acid is a product of the pentose phosphate pathway, and its abnormally high levels in tissues can cause metabolic acidosis. And the pentose phosphate pathway can promote the production of reducing equivalents, such as NADPH, which will be used for reducing biosynthesis reactions in cells (Sun et al., 2017; Li Z. et al., 2019). Based on the previous results, our current results suggests that mice from the abdominal irradiation group may have a certain degree of hyperoxidation, which is consistent with the previously reported bystander effect of radiotherapy.

Arginosuccinic acid is synthesized from citrulline and aspartic acid and is used as the precursor of arginine in the urea cycle or citrulline-NO cycle. Arginosuccinate is the precursor of fumaric acid produced by argininosuccinate lyase in the citric acid cycle. The reduction of fumaric acid and malic acid was reported to be associated with right ventricular remodeling in severe pulmonary hypertension rats (Sakao et al., 2021), suggesting that arginine succinate utilization disorder may ultimately affect energy metabolism and ventricular remodeling. Guanidinoacetate (GAA) is involved in the metabolism of glycine, serine and threonine, the metabolism of arginine and proline, and the production of creatine. The main metabolic function of creatine is producing creatine phosphate through the enzyme creatine kinase and phosphate group, which leads to ATP regeneration. Therefore, we speculate that the increase in myocardial guanidinoacetate will result in an increase in the production of creatine, and creatine utilization disorders could eventually affect the energy supply of the heart.

L-5-hydroxytryptophan is an aromatic amino acid produced from the essential amino acid l-tryptophan. When 5-hydroxytryptophan levels were high enough, it could be a neurotoxin and a metabolic toxin. It is reported that long-term use of L-5-HTP provides significant protection against the development of deoxycorticosterone acetate-salt-treated (DOCA)-induced hypertension and cardiac hypertrophy in rats (Fregly et al., 1987). Long-term-use of L-5-HTP can effectively reduce the increase in blood pressure in the DS model (Baron et al., 1991). In addition, L-5-hydroxytryptophan can also increase renin activity (Barney et al., 1981), thereby affecting the structure and function of the heart. Therefore, the increase in L-5-hydroxytryptophan in the AIR group observed in this study could be a compensatory protective effect or an unclear toxic effect. Tryptophan could generate indole-3-acetic acid (IAA) (Leveau and Gerards, 2008), and IAA degrading is involved in tricarboxylate cycling, gluconeogenesis, and carbon/nitrogen sensing (Leveau and Gerards, 2008), which may induced the cardiac dysfunction in AIR group. Lysine (Lys) is an essential amino acid that not only participates in protein synthesis, but also promotes the cross-linking of collagen peptides. Lysine is also often involved in histone modification and therefore affects the epigenome. According to reports, the acetylation of histone lysine is associated with myocardial remodeling (Peng et al., 2021; Han et al., 2022), and the lysine methylation of histone promotes the reprogramming of myocardial cells by fibroblasts (Singh et al., 2021). Meanwhile, histone lysine-specific demethylase one induced renal fibrosis (Dong et al., 2022; Zhang et al., 2022). However, a large increase in lysine was observed in the abdominal radiation group of this study, combined with the enrichment of other metabolites concentrated in the lysine metabolic pathway, further indicating that the large accumulation of lysine, but its role in the changes in cardiac structure and function is still need further verification.

Abnormalities in amino acid metabolism could be implicated in cardiac dysfunction and remodeling. However, the upstream and downstream regulatory mechanisms need to be further explored. Through KEGG pathway analysis, we speculate that some pathways may lead to changes in small molecular metabolites, and ultimately lead to abnormal cardiac function and remodeling. Our research found that differential metabolites are mainly enriched in HIF-1 signaling pathway, which participates in the regulation of many biological processes related to metabolic differences (Denko, 2008), especially oxygen homeostasis. HIF-1 is a switch that controls the recognition and response of cells to changes in oxygen status. HIF-1 controls oxygen utilization by regulating glucose metabolism and redox homeostasis (Semenza, 2014; Zhang et al., 2019). Radiation can activate the HIF-1 signaling pathway by damaging endothelial cells, and regulate vascular endothelial growth factor (VEGF) and CXCL12 (C-X-C motif chemokine 12) to increase the effect of killing target cells (Meijer et al., 2012; Sun et al., 2019; Huang and Zhou, 2020). For normal cell metabolism, radiation-dependent HIF1α converts oxidative phosphorylation to glycolysis and increase radiation resistance (Lall et al., 2014). However, this study found that irradiation of the abdomen of mice activated the cardiac HIF-1 pathway. Whether this change protects or damages the cardiac response related to the bystander phenomenon requires further research. Besides, the mechanism of HIF-1 activation also needs further study.

### Limitations

Although this study revealed that long-term cardiac damage caused by remote abdominal irradiation is related to metabolic remodeling, especially amino acid metabolic remodeling, and downstream involving apoptosis, it did not clarify the specific pathway mechanism. Meanwhile, this study did not measure RV function, electrocardiogram and baseline echocardiographic parameters.

## Conclusion

**FIGURE 7:**
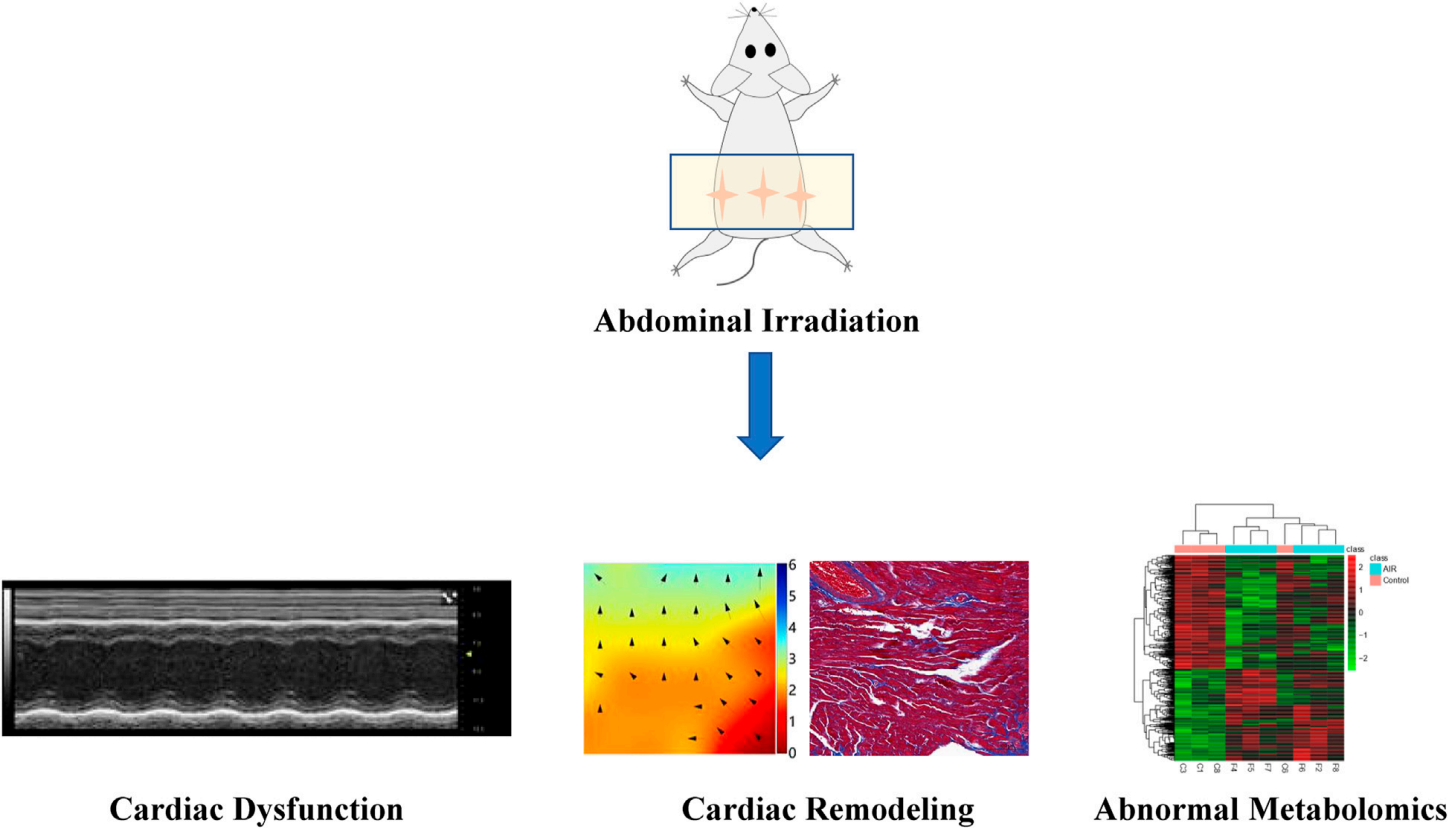
Schematic image depicts long-term cardiac damage associated with abdominal irradiation in mice.

Abdominal irradiation causes long-term damage to the non-irradiated heart, as reflected by electrical and structural remodeling and mechanical dysfunction associated with abnormal amino acid biosynthesis and metabolism (Figure 7).

## Data Availability

The original contributions presented in the study are included in the article/supplementary materials, further inquiries can be directed to the corresponding authors.
